# Clinical Characteristics and Management of Functional Pancreatic Neuroendocrine Neoplasms: A Single Institution 20-Year Experience with 286 Patients

**DOI:** 10.1155/2020/1030518

**Published:** 2020-11-06

**Authors:** Yuqing Qu, Haoming Li, Xianling Wang, Yulong Chen, Qinghua Guo, Yu Pei, Jin Du, Jingtao Dou, Jianming Ba, Zhaohui Lv, Yiming Mu

**Affiliations:** ^1^Department of Endocrinology, Chinese PLA General Hospital, Beijing 100853, China; ^2^Department of Endocrinology, Yantai Yuhuangding Hospital, Yantai 264000, Shandong Province, China; ^3^Department of Endocrinology, Shexian Hospital, Handan 056400, Hebei Province, China

## Abstract

**Background:**

Functional pancreatic neuroendocrine neoplasms (PanNENs) are very rare disorders but have complex spectrum, including insulinoma, gastrinoma, glucagonoma, somatostatinoma, and VIPoma. Patients with PanNENs usually present with characteristic symptoms caused by corresponding hormone hypersecretion. It has always been challenging in dealing with such rare but complicated disorders. In this report, we analyzed the clinical characteristics of functional PanNENs in a large cohort of Chinese patients and summarized our clinical experience in diagnosis and treatment.

**Methods:**

The retrospective analysis was performed in patients with a definite diagnosis of functional PanNENs hospitalized in Chinese PLA General Hospital between 2000 and 2020. The clinical characteristics, surgical information, and pathological findings were extracted from their medical records and were analyzed.

**Results:**

Totally, 286 patients (gender: male 103 and female 183; age: 45.55 ± 15.23 years old) were diagnosed with definite functional PanNENs. The most frequent functional PanNENs were insulinoma (266/286) followed by glucagonoma (10/286), somatostatinoma (3/286), adrenocorticotropic hormone- (ACTH-) producing tumor (3/286), gastrinoma (2/286), and VIPoma (2/286). Nine patients were diagnosed with multiple endocrine neoplasia type 1 (MEN1) in which all the associated functional PanNENs were insulinomas. The duration from symptoms' onset to confirmed diagnosis was 3.67 ± 4.28 years. Two hundred and eighty patients with tumor localized in pancreatic or with limited metastasis underwent surgery. The symptoms associated with hormonal oversecretion were improved significantly after surgery. Five patients with unresectable metastases or tumor recurrence after surgery were administrated with systemic chemotherapy or other targeted therapies. With these various therapies, the symptoms were also partially relieved. According to findings in pathological and immunochemical examination, all the functional PanNENs were categorized into NEN-G1 (41.95%), NEN-G2 (54.90%), NEN-G3 (3.15%), and NEC-G3 (0%).

**Conclusion:**

Patients with suspected functional PanNENs should have a systematic endocrine examination at diagnosis. Multidisciplinary collaborations are essential for precise diagnosis and tumor localization. A successful surgery or other targeted therapies can improve the prognosis of patients with such rare but complex disorders.

## 1. Introduction

Pancreatic neuroendocrine neoplasms (PanNENs) are usually classified as functional PanNENs and nonfunctional PanNENs. Patients with functional PanNENs display symptoms corresponding to neuroendocrine hormones excessively released by these neoplasms. The most frequent functional PanNENs are insulinoma followed by glucagonoma, somatostatinoma, gastrinoma, and VIPoma. Some other rare neoplasms can secrete adrenocorticotropic hormone (ACTH)/corticotropin-releasing hormone (CRH) or parathyroid hormone-related protein (PTH-RP), causing ectopic hormone secretion syndrome. Nonfunctional PanNENs do not secrete hormone or secrete some peptides and therefore do not cause specific symptoms [1].

The functional PanNENs are rare and can have complex presentations. In clinical practice, we have observed that the misdiagnosis and delayed therapy about this disorder are very common. It has always been a challenge for endocrinologists in dealing with such special and complicated disorders. In China, there are few large series of studies about the prevalence and comprehensive clinical spectrum of functional PanNENs [2]. In this report, we analyzed the clinical characteristics of functional PanNENs in a large Chinese cohort admitted in our hospital and summarized our clinical experience in diagnosis and therapy.

## 2. Materials and Methods

### 2.1. Patients

Patients with confirmed diagnosis of functional PanNENs between January 2000 and January 2020 were identified through the Chinese PLA General Hospital medical database. With a standardized data extraction form, we obtained the medical records associated with the diagnosis and therapy of functional PanNENs for the patients.

### 2.2. Methods

All the complex clinical information was retrospectively analyzed, including (1) general information, such as gender, age, family history, and presence of symptoms; (2) tumor localization determined with various methodologies, including ultrasound, abdominal enhanced computed tomography (CT), magnetic resonance imaging (MRI), digital subtraction angiopraphy (DSA) of superior pancreaticoduodenal artery, ^18^F-FDG PET (positron emission tomography)/CT, and ^68^Ga-DOTA-TATE PET/CT. The invasive imaging studies included selective angiography, endoscopic ultrasound (EUS), and intraoperative ultrasound (IOUS); (3) tumor features including number, size, distribution, location, and metastasis; (4) treatments including surgery, chemotherapy, somatostatin analogues, and interventional approaches; and (5) pathological and immunohistochemical assessment of tumors [3].

The diagnosis of functional PanNENs included multiple criteria including (1) the presence of symptoms and/or signs caused by pancreatic neuroendocrine hormones, such as repeated hypoglycemia for insulinoma, serious and recurrent multiple peptic/duodenal ulcers for gastrinoma, necrolytic migratory erythema (NME) for glucagonoma, and watery diarrhea for VIPoma; (2) laboratory evidence of the excessive hormone production in patients. For example, the diagnosis of insulinoma was facilitated by the presence of hyperinsulinemic hypoglycemia (plasma glucose concentrations <2.8 mmol/L; meanwhile, plasma insulin concentrations ≥3 *μ*U/mL). Regrettably, other pancreatic neuroendocrine hormones except insulin could not be measured previously in our hospital; (3) localization of tumors in pancreases determined by multiple imaging tests; (4) confirmation of neuroendocrine tumors by pathological or/and immunohistochemical examination of tumor samples from biopsy or surgery; and (5) relief of respective clinical symptoms after tumor removal via surgery or targeted therapies [4].

All the PanNENs in this series were categorized according to the 2017 World Health Organization (WHO) Classification, in which NET (well-differentiated tumor) and NEC (poorly differentiated) are distinguished by tumor differentiation. NET is further divided based on the mitotic count or Ki-67 index as NET-G1 (mitotic index (MI) <2/high-power fields (HPFs), Ki-67 index <3%), NET-G2 (MI 2–20/HPFs, Ki-67 index 3–20%), or NET-G3 (MI >20/HPFs, Ki-67 index >20%).

A diagnosis of multiple endocrine neoplasia type 1 (MEN1) was established in patients with one of the three criteria: (1) clinical features (two or more of the associated tumors in an individual); (2) familial history (diagnosis of at least one type of tumors in a first-degree relative); and (3) a germline mutation [1].

This study protocol was approved by the Institutional Review Board of Chinese PLA General Hospital and was in accordance with the ethical guidelines for epidemiological research.

### 2.3. Statistical Analysis

Continuous data were presented as mean ± standard deviation (SD). Statistical analyses were performed using IBM SPSS Statistics version 22.0 (Chicago, IL, USA).

## 3. Results

### 3.1. General Information

A total of 286 patients (male 103; female 183) were diagnosed with functional PanNENs between January 2000 and January 2020. The mean age was 45.55 ± 15.23 years old. The most frequent functional PanNENs were insulinoma (266/286) followed by glucagonoma (10/286), somatostatinoma (3/286), ectopic ACTH-producing tumor (3/286), gastrinoma (2/286), and VIPoma (2/286). Nine patients with insulinoma were finally diagnosed to have MEN1 according to the diagnostic criteria. On the basis of findings in pathological and immunochemical examination, all the functional PanNENs were categorized to NEN G1 (41.95%), NEN G2 (54.90%), NEN G3 (3.15%), and NECG3 (0%). For all cases, the duration from the onset of primary symptoms to final diagnosis confirmation in our hospital was 3.67 ± 4.28 years (Table 1).

### 3.2. Insulinoma

Two hundred and sixty-six patients who presented with severe and recurrent hypoglycemia symptoms were diagnosed to have insulinoma, including 250 patients with single tumor and 16 patients with multiple tumors. Among these patients, 257 had sporadic tumor, and 9 patients had MEN1. When hypoglycemia (plasma glucose concentration: 1.89 ± 0.59 mmol/L (normal range: 3.4–6.1 mmol/L)) attacked in fasting time or 72 h fasting glucose test, plasma insulin concentration ((39.59 ± 30.14) mU/L, normal range: (2.6–24.9 mU/L)) and insulin-releasing index ((1.23 ± 1.05) mIU/L/(mg/dL), normal range: <0.3 (mIU/L)/(mg/dL)) were much higher. Sometimes, the presentations of the hypoglycemic episode mimic epilepsy seizure in many patients. Forty-four patients (16.54%) with insulinoma had ever been misdiagnosed to have epilepsy before hospitalization and prescribed with antiepileptic drugs (AEDs) for 1–10 years.

Some of the noninvasive and invasive imaging studies were performed in tumor localization. The performance of ultrasound examination, CT scan, MRI scan, EUS, contrast-enhanced ultrasound examination, DSA, ^18^F-FDG PET/CT, and ^68^Ga-DOTA-TATE PET/CT was 66.2% (176/266), 80.5% (214/266), 53.0% (141/266), 52.3% (139/266), 46.6% (124/266), 57.9% (154/26), 64.51% (12/266), and 1.50% (4/266), respectively. And the positive detection rate was 27.3% (48/176) with ultrasound examination, 76.2% (163/214) with CT, 83.8% (129/155) with DSA, 87.1% (108/124) with contrast-enhanced ultrasound examination, 92.9% (131/141) with MRI, 96.4% (134/139) with EUS, 83.33% (10/12) with ^18^F-FDG PET/CT, and 75.0% (3/4) with ^68^Ga-DOTA-TATE PET/CT, respectively. In our hospital, a patient can undergo surgery or biopsy only after the tumor is localized with three or more preoperative localization tests.

In this cohort, 263 patients underwent operation. Two hundred and forty-seven patients with single tumor and 16 patients with multiple tumors were cured with one surgery. Three patients had undergone one exploratory surgery, and two patients had undergone two exploratory surgeries in other hospitals before tumors could be precisely located. Unfortunately, the insulinomas were not detected in surgical exploration, and the hypoglycemia could not be relieved. After admitted to our hospital, all these patients were found to have small insulinomas by multiple diagnostic methods. These tumors subsequently were resected successfully. Another one patient could not go through the treatment because of poor healthy condition.

For other patients with unresectable metastatic disease, medical management is reserved. Diazoxide and transarterial embolization/chemoembolization were carried out in 2 cases, respectively. Three patients with progressive insulinomas were prescribed with long-acting somatostatin analogue after debulking surgery. With these therapies, the frequency and severity of hypoglycemia events reduced significantly.

### 3.3. Gastrinoma

Three patients suffered from recurrent and multiple peptic/duodenal ulcers. They had been prescribed with proton-pump inhibitors (PPIs) for 2 years, but with unsatisfactory results. After admission, the diagnosis of gastrinomas was presumed even without gastrin examination. Later, the suspected neoplasms were detected in these patients with various methods which have been described above. One patient in poor health condition refused biopsy and operation, and no pathological findings could be obtained. So, this patient was not enrolled in this series. The other two patients underwent successful surgery, and the positive findings of gastrin in immunochemical examination confirmed the presumed diagnosis. No peptic ulcers recurred later.

### 3.4. Glucagonoma

Ten patients were diagnosed with glucagonoma in this cohort. Three patients initially presented with necrolytic migratory erythema (NME), including 2 patients with diabetes mellitus (DM) and 1 patient with gallstones. Two patients had modest DM at diagnosis. Five patients presented with abdominal pain and/or pancreatic tumor.

Five patients underwent surgery. After the tumor was resected, the symptoms of NME completely relieved in 3 patients without distant metastasis and partially relieved in other 2 patients with metastasis. The ultrasound-guided microwave ablation therapy was performed in one patient with multiple distant metastasis.

### 3.5. Somatostatinoma

Three patients were diagnosed with somatostatinoma. Two patients presented with DM and 1 patient with cholelithiasis. All 3 patients underwent surgery. One patient with distant metastases was administrated with long-acting octreotide microsphere injection after debulking surgery.

### 3.6. VIPomas

Two patients presented with unknown recurrent diarrhea for 1 year. They were suspected to have somatostatinoma after the tumors were detected in pancreas, even without VIP examination. Both patients underwent surgery. Immunohistochemical staining showed intense immunoreactivity of VIP. One patient with liver metastases was later administered with transarterial embolization/chemoembolization. The symptoms of recurrent diarrhea in these two patients were clearly relieved after therapy.

### 3.7. ACTH-Producing Tumor

Three patients presented with progressive Cushing's syndrome. The concentrations of plasma ACTH ((74.23 ± 29.35) pmol/L (<10.12 pmol/L at 8 am)) and serum free cortisone ((1348.16 ± 422.74) nmol/L (198.7–797.5 nmol/L at 8 am)) were both at very high levels. Subsequently, high-dose dexamethasone suppression tests confirmed the diagnosis of ectopic ACTH syndrome (EAS). Tumors in the pancreas were localized which was believed to be the pathogen. After surgery, Cushing's symptom relieved significantly, and also, the elevated plasma ACTH concentration and serum free cortisone decreased significantly. Immunohistochemical stains showed intense immunoreactivity of ACTH.

### 3.8. Multiple Endocrine Neoplasia Type 1 (MEN1)

In this cohort, 9 patients were diagnosed with MEN1 according to the diagnosis criteria described in Methods. All of the MEN1-associated functional PanNENs were insulinomas (6 single and 3 multiple). These 9 patients underwent surgery. Seven patients were cured. Two patients were cured after the second surgery after having relapse hypoglycemia.

Gene sequencing was performed in 7 patients. The MEN1 gene mutations were detected in exon 2 (c.358-360delAAG, c.248-251delTGTC, c.234-252del9, and c.416A ＞ G heterozygous mutation) and exon 3 (c.1150delG, c.512G22, and c.593G ＞ A heterozygous mutation).

## 4. Discussion

Functional PanNENs are uncommon tumors in the pancreas, with a diverse group of tumors which show heterogeneity in pathologic, functional, and clinical features. Insulinoma is the most frequent functional PanNEN with an incidence of 1–4/million people/year. Other tumors such as gastrinoma, glucagonoma, somatostatinoma, and VIPoma are even more rarer. Because the incidence of these neoplasms is very low and the presentations are complex, the diagnosis has always been delayed for long time. Most of the functional PanNENs except insulinoma have more malignant potential. It is reported that distant metastasis has always occurred before the diagnosis is confirmed, which has worse prognosis.

To our knowledge, this is a larger cohort of functional PanNENs in China. In the present study, we summarized the clinical presentations, diagnosis, locations, and therapies of 286 patients with various functional PanNENs hospitalized in our hospital between January 2000 and January 2020. All these findings may provide more valuable information for multidisciplinary collaboration in dealing with this complex disorder [1].

### 4.1. Presentation of Functional PanNENs

The major symptoms of insulinoma are associated with the effects of hypoglycemia on the CNS, such as confusion, headaches, visual disturbances, behavioral changes, and even recurrent coma. Most patients also have symptoms due to adrenergic stimulation caused by hypoglycemia, such as sweating, tremor, palpitations, and irritability. However, hypoglycemic symptoms sometimes are similar with epilepsy seizure. In this cohort, 16.48% patients with insulinoma had once been misdiagnosed to have epilepsy and incorrectly prescribed with AEDs for long time, which should be emphasized in differential diagnosis [5].

Gastric hypersecretion can cause recurrent peptic ulcers, which is known as Zollinger–Ellison syndrome (ZES). The diagnosis of ZES and gastrinoma should be presumed in patients with recurrent peptic ulcers, multiple ulcers, or ulcers in unusual positions such as the proximal jejunum or distal duodenum [6]. The suspicion of gastrinoma has always been overlooked or delayed due to regular PPI therapy, which can have the symptoms relieved. Most gastrinomas are sporadic (75%–80%), and approximately 20–25% are associated with MEN1.

Glucagonoma usually presents as a sporadic neoplasm, but in 51–78% patients, it has already developed to distant liver metastasis at the time of diagnosis. NME is a hallmark clinical sign of glucagonoma and is present in approximately 70% patients with glucagonoma. The patients always consult in the dermatological department and could not get the correct diagnosis in time. Mild DM is another common presentation. In this report, 10 patients were diagnosed with glucagonoma, whose chief presentations included NME, DM, or gallstones.

Somatostatinoma may be sporadic (93.1%) or associated with neurofibromatosis type 1 (NF1), MEN1, and Von Hippel–Lindau syndromes (6.9%) [7]. In most patients (92.7%), the clinical somatostatinoma syndrome was caused by the inhibition of insulin, glucagon, gastrin, secretin, and somatotrophin. This “inhibitory” syndrome consists of DM, diarrhea/steatorrhoea, and cholelithiasis. The diagnosis has been difficult because the symptoms are generally nonspecific and mild. In this cohort, 2 patients with somatostatinoma presented with DM, and 1 patient presented with cholelithiasis.

VIPomas can cause increased intestinal secretion of Na^+^, K^+^, HCO_3_^−^, and Cl^−^, as well as bone resorption, vasodilation, and inhibition of gastric acid section. These effects lead to a well-defined clinical syndrome characterized by watery diarrhea, hypokalemia, and hypochlorhydria [8]. In this cohort, 2 patients with recurrent diarrhea were diagnosed to have definite somatostatinoma.

It is important to point out that three patients in our cohort had EAS caused by PanNENs. EAS is a very rare disorder, accounting for 15% in Cushing's syndrome. PanNENs rarely produce ACTH and cause Cushing's syndrome. Usually, the differential diagnosis of EAS caused by pathogenic tumors in pancreas is very difficult. Most of the EAS-PanNENs have distant hepatic metastases at the time of diagnosis, with rapid progression and poor prognosis. As described in these 3 patients, the mean time from the symptom onset to definite diagnosis was only 8.67 ± 13.28 months.

### 4.2. Localization of Functional PanNENs

Localizations of tumors with various techniques are required after the diagnosis of functional PanNENs is clinically and biochemically confirmed. Accurate localization of tumors is vital for detecting primary tumors and tumor extensions, as well as for the surgical resection.

In recent years, numerous localization studies have been performed in clinical practice. The noninvasive imaging studies include ultrasound, CT, MRI, ^18^F-FDG PET/CT, and ^68^Ga-DOTA-TATE PET/CT, but sometimes, the tumors are too small and occult (<1 cm) to be detected easily with one or more traditional noninvasive techniques.

Since insulinomas and other PanNENs are vascular tumors, selective angiography is usually positive in 60%. The invasive imaging studies include selective angiography, functional localization methods (angiography with secretion or calcium stimulation and evaluation of hepatic venous gastrin gradients by portal venous sampling), EUS, and IOUS. However, some of these invasive or novel techniques have not been performed in our hospital.

In our cohort, the positive detection rate of insulinoma was 27.3% with ultrasound examination, 76.2% with CT, 83.8% with DSA, 87.1% with contrast-enhanced ultrasound examination, 92.9% with MRI, and 96.4%with EUS, respectively. Because of economic limitations, PET^18^F-FDG PET/CT and ^68^Ga-DOTA-TATE PET/CT were only carried out in a few patients with suspected insulinomas in recent years. The efficacy was satisfactory (10/12 patients and 3/4 patients, respectively) in detecting small and occult tumors.

### 4.3. Management of Functional PanNENs

#### 4.3.1. Surgery

Surgery has been the primary and most important treatment for functional PanNENs and also the only possible curative procedure. Patients who undergo surgery have better prognosis compared to those who undergo other nonsurgical treatments.

Patients with functional PanNENs without distant organ or lymph node metastases could be cured completely by surgery alone, so surgical resection should be recommended in these patients. Liver is the most common site of distant metastases followed by lymph nodes, bone, and mesentery/omentum/peritoneum. When metastasis is limited to the liver and complete resections of primary and metastases are available, curative surgery should also be applied. When tumor shows local regional infiltration and/or invading adjacent organs and complete resection of metastatic lesions is impossible, debulking surgery is often indicated [9–15].

In this cohort, most patients with single or multiple tumors localized in the pancreas underwent one-time successful surgery. Only a few patients had to undergo a second surgery because of recurrence. The symptoms associated with hormone oversecretion completely relieved after curative surgery or partially relived after debulking surgery. All the functional PanNENs were categorized into NEN G1 (41.38%), NEN G2 (55.52%), NEN G3 (3.10%), and NECG3 (0%).

#### 4.3.2. Systemic Chemotherapy

Systemic chemotherapy is administered to patients who have rapid disease progression, tumor with aggressive pathological characteristics, and high proliferative indexes.

Streptozocin-based chemotherapy regimens have been a standard treatment of PanNENs for several decades, with significant improvement in progression-free survival (PFS) and overall survival (OS). Everolimus is an oral mammalian target of the rapamycin (mTOR) inhibitor which shows to be effective in patients with clinically progressive PanNENs. Most of functional PanNENs are highly vascular cancers. They frequently express the vascular endothelial growth factor (VEGF) ligand and its receptors. Sunitinib is a novel, oral, multitargeted tyrosine kinase inhibitor with antiangiogenic and antitumor activities, reported to have a significant PFS benefit in progressive PanNENs [15, 16].

#### 4.3.3. Other Therapies

Over 70% of functional PanNENs express variable levels of somatostatin (SST) receptors on their cell surface. Somatostatin is a peptide hormone that can bind to SSTR subtypes 1–5 and inhibit the secretion of other pancreatic neuroendocrine hormones. Long-acting somatostatin analogues have been shown to be very effective for alleviating hormonal symptoms. Also, it can inhibit the tumor growth [15]. In this cohort, the usage of long-acting somatostatin analogue was not common. Three patients with progressive insulinomas and one patient with progressive somatostatinoma were prescribed with long-acting somatostatin analogue after debulking surgery.

Radiolabeled SSA therapy is a new form of targeted radiotherapy, which delivers radioactive isotopes to SSTR-expressing functional PanNENs. In clinical practice, the novel therapy has been shown to effectively alleviate symptoms in patients [16].

The liver is the dominant site of metastases for functional PanNENs. Hepatic artery chemoembolization (TAE) or radioembolization is recommended for unresectable liver metastases [15]. In this cohort, a few patients with liver metastasis underwent this kind of therapy.

Radiofrequency ablation (RFA) and microwave ablation therapy have been novel and effective techniques for patients with functional PanNENs who are not eligible for surgery. They have showed satisfactory effects in reducing tumor volume and eliminating symptoms associated with hormone oversecretion. In this cohort, one patient with progressive glucagonoma successfully underwent microwave ablation therapy.

Diazoxide is an effective drug for controlling hypoglycemia though direct action on the islet beta-cells. In this cohort, one patient with insulinoma and distal metastases was prescribed with diazoxide and had hypoglycemia symptoms partially relieved.

In this cohort, 3 cases with EAS caused by PanNENs were diagnosed. Cushing's syndrome was quickly relieved after PanNENs were resected. Unfortunately, EAS relapsed soon after, which was in agreement with the previous report in the literature (citations). It has been reported that steroid synthesis inhibitors (metyrapone, mitotane, trilostane, ketoconazole, and aminoglutethimide), glucocorticoid receptor antagonists (mifepristone), and adrenal-directed medical therapies are also useful in controlling excess cortical. Palliative bilateral suprarenalectomy is also recommended to control Cushing's symptoms.

### 4.4. Management of MEN1

MEN1 is an autosomal-dominant tumor syndrome with an approximate prevalence of 1/30,000 people. Germline heterozygous mutation in the MEN1 tumor suppressor gene encoding menin predisposed to tumor development. This disorder consists of the development of multiple neuroendocrine tumors in the patient, principally at parathyroid glands, anterior pituitary, and gastroenteropancreatic (GEP) tracts.

Nearly 80% of the patients with MEN1 have multiple PanNENs in the pancreas and/or the duodenum. The most frequent functional PanNENs are insulinoma and gastrinoma. In this cohort, 9 patients were diagnosed with MEN1, and the MEN1-associated functional PanNENs were well-differentiated insulinomas. Seven patients were confirmed with genetic mutations in MEN1. Various gene mutations of MEN1 were detected in exon 2 and exon 3.

All the patients underwent pancreas surgery and parathyroid resection, at the same time or at different times. The recurrent hypoglycemia symptoms associated with insulinoma got relieved after a complete insulinoma resection.

## 5. Study Limitations

This was a retrospective medical record review with all its inherent biases. Previously, the availability of localization methods has been limited, and the accuracy has not been very satisfactory. In the past 10 years, several new technologies have been used in localization, by which small or occult PanNENs could be detected more efficiently. Other pancreatic neuroendocrine hormones except insulin could not be measured previously in our hospital. In this series, not all the MEN1 patients in our study had MEN1 gene mutations investigated.

## 6. Conclusion

From our experiences with successful treatment of the large cohort of patients with functional PanNENs in China, we advocate that all patients with suspected PanNENs should have a systematic endocrine examination at diagnosis. With more promising diagnostic techniques and therapeutic methods emerging in recent years, multidisciplinary cooperation will provide better prognosis for patients with such rare but complex disorders.

## Figures and Tables

**Table 1:** Summary of the reported cases of functional pancreatic neuroendocrine tumors.

	Gender (male/female)	Age	Duration	Size						Grade				Number		Heredity	
				Single			Multiple										
				≤1 cm	1-2 cm	≥2 cm	≤1 cm	1-2 cm	≥2 cm	NET-G1	NET-G2	NET-G3	Unknown	Single	Mutiple	Sporadic	MEN1
Total	103/183	45.55 ± 15.23	3.67 ± 4.28 (y)	25	179	64	5	9	4	120	157	9		268	18	277	9
Insulinoma	95/171	45.55 ± 15.25	3.86 ± 4.34 (y)	22	178	50	4	9	3	113	145	8		250	16	257	9
Glucagonoma	4/6	51.2 ± 13.58	1.54 ± 2.35 (y)	3		7				3	7			10	0	10	0
Somatostatinoma	1/2	48.33 ± 16.94	0.77 ± 0.33 (y)		1	1			1	3				2	1	3	0
ACTH-producing tumor	2/1	27.67 ± 8.5	8.67 ± 13.28 (m)			2	1				2	1		2	1	3	0
Gastrinoma	0/2	46 ± 7	1.02 ± 0.98 (y)			2				1	1			2	0	2	0
VIPoma	1/1	38.5 ± 3.5	1 (y)			2					2			2	0	2	0

## Data Availability

All data generated or analyzed during this study are included within the article.
